# New Class of S-Pseudo Bounded Modules With Some Related Concepts

**DOI:** 10.12688/f1000research.172196.1

**Published:** 2025-12-09

**Authors:** Amal Madhi Rashid, Buthyna Najad Shihab

**Affiliations:** 1 Mathematics, University of Baghdad College of Education for Pure Science Ibn Al-Haitham, Baghdad, Baghdad Governorate, 31001, Iraq; 2 Mathematics, University of Baghdad College of Education for Pure Science Ibn Al-Haitham, Baghdad, Baghdad Governorate, 10001, Iraq

**Keywords:** S-Pseudo bounded module, Fully polyform module, Multiplication module, Critically compressible module, Scalar module.

## Abstract

In the present study , every module M is unitary and every ring F is commutative with identity. We gave a definition of a new class F−module which is namely S-pseudo bounded module symbolically (S-PS.B. F− module) and introduced some different approaches to attach this class with other types of well-known modules such that monoform module, quasi-Dedekind module, compressible module and retractable module. The main purpose of this article is to present a few new conditions for some corollaries and properties. The F− homomorphism of monoform and compressible modules connect in a useful way with an endomorphism of a F− module M that we relied on it in the definition of S-pseudo bounded module. We used the symbol End(M) which means the set of all endomorphism maps of F− module M . Also S-pseudo bounded module gave us directly or with some conditions different modules such as retractable module, an injective module and others.

## 1. Introduction

The notion of bounded module was studied by Carl Faith where (if there exists x∈M such that ${\mathrm{ann}}_{F}\left(M\right)={\mathrm{ann}}_{F}\left(x\right)$ then M is said to be bounded). ^1^ Moreover, the concept of bounded submodule introduced with details by AL-LNI where (if there exists x∈ℵ such that ${\mathrm{ann}}_{F}\left(ℵ\right)={\mathrm{ann}}_{F}\left(x\right),$ then ℵ is said to be bounded submodule of M ). ^2^ The concept of almost bounded submodule was submitted by Buthyna Najad where (if there exists an element x∈M,x∉ℵ such that ${\mathrm{ann}}_{F}\left(ℵ\right)={\mathrm{ann}}_{F}\left(x\right)$ then ℵ is called almost bounded submodule). ^3^ Also, scalar modules and prime modules are involved in several properties as a condition to attach S-Pseudo bounded module with other modules. Note that if ${\mathrm{ann}}_{F}\left(ℵ\right)={\mathrm{ann}}_{F}\left(x\right),$ for every submodule ℵ of M then M is said to be prime module. ^4^ Furthermore, if M is finitely generated , then M is compressible F−module if and only if it is uniform and prime. ^5^ This study investigates how S-PS.B. modules relate to other well-known module types offering new insights into these connections. We explore how these modules relate to monoform modules, compressible modules, critically compressible modules and Rickart modules directly or with some conditions.

In this article, we investigate a new class of module called S-Pseudo bounded module where in Section 2 some related facts are reviewed and Section 3 contained some definitions with examples while in Section 4 we introduced some significant relationships and connected with other modules.

## 2. Preliminaries

Definision 2.1 ^1^A F−module M is bounded module if there exists an element x∈M such that ${\mathrm{ann}}_{F}\left(M\right)={\mathrm{ann}}_{F}\left(x\right).$

Definision 2.2 ^6^A F−module M is said to be scalar if for every φ∈End(M) there exists r∈F such that φ(x)=rx,∀x∈M.

Definision 2.3 ^7^A F−module M is called monoform if ∀ℵ≠0 of M is dense where a submodule ℵ of M is dense if for any x,y∈M,x≠0 there exists t∈F such that ty∈ℵ and tx≠0 .

Equivalently, A F−module M is said to be monoform if ∀ℵ≠0 of M and ∀0≠φ∈Hom(ℵ,M) then φ is monomorphism. ^7^ Also , if M is monoform module, then it is uniform and prime and hence we deduce that ${\mathrm{ann}}_{F}\left(M\right)$ is prime ideal of F.

Definision 2.4 ^8^A F−module M is said to be finitely annihilated F−module if there exists a finitely generated submodule ℵ of M such that ${\mathrm{ann}}_{F}\left(M\right)={\mathrm{ann}}_{F}\left(ℵ\right).$

Definision 2.5 ^9^A F−module M is called quasi-Dedekind if ∀ℵ≠0 of M is quasi-invertible where ℵ is quasi-invertible if Hom(M/ℵ,M)=0.

Equivalently, a F−module M is said to be quasi-Dedekind if for each non-zero endomorphism of M is a F−monomorphism.

Definision 2.6 ^10^ A F−module M is called polyform if every essential submodule ℵ of M is dense. Note that every monoform is polyform.

Definision 2.7 ^10^A F−module M is said to be fully polyform if every P-essential submodule of M is dense where ℵ is called P-essential if every pure submodule k of M such that ℵ∩k=(0) implies that k=(0).

Definision 2.8 ^10^A F−module M is said to be fully retractable if for every non-zero submodule ℵ of M and each non-zero homomorphism f∈Hom(ℵ,M) implies that Hom(ℵ,M)≠0.

Definision 2.9 ^11^A F−module M is called coprime if ${\mathrm{ann}}_{F}\left(M\right)={\mathrm{ann}}_{F}\left(M/ℵ\right)$ for every proper submodule ℵ of M.

Corollary 2.10 ^6^If M is a multiplication finitely generated F−module, then M is a scalar F−module.

Remark 2.11 ^6^Let M be an injective scalar F−module . Then ℵ is a scalar submodule of M.

Remark 2.12 ^12^Every finitely generated F−module is finitely annihilated.

Proposition 2.13 ^12^Let M be a multiplication F−module. Then M is finitely generated if and only if M is finitely annihilated.

Proposition 2.14 ^6^Let M be a scalar torsion-free F−module with ( F is an integral domain). Then every φ∈End(M) is F−monomorphism.

Proposition 2.15 ^13^Let M be a quasi-Dedekind retractable F−module. If every 0≠φ∈End(M) is a monomorphism, then M is compressible module.

Proposition 2.16 ^14^Let M be a retractable F−module such that End( M) is a domain. Then M is critically compressible module if and only if it is polyform.

Proposition 2.17 ^14^Let M be a retractable F−module. Then M is critically compressible module if and only if every non-zero partial endomorphism of M is monomorphism.

Proposition 2.18 ^14^Let M be a fully retractable F−module with End( M) is a domain. Then M is polyform.

Definition 2.19 ^15^A F−module M is called Rickart F−module if and only if Kerφ is a direct summand of M.

Proposition 2.20 ^15^If M is an injective prime F−module, then M is Rickart module.

Definition 2.21 ^16^A F−module M is called N-Rickart if for every homomorphism g:M→ℵ,kerg is a summand of M.

Corollary 2.22 ^14^If M is uniform module, then M is fully polyform if and only if M is monoform module.

Corollary 2.23 ^17^If M is finitely generated F−module , then M is compressible F−module if and only if M is uniform prime module.

## 3. S-Pseudo bounded modules

In this part, a new class of an F−module will be investigated with some definitions and examples related to S-PS.B. F -module which depends on an endomorphism map over an F−moduleM.

Definition 3.1 A proper submodule ℵ of a F−moduleM is called S-pseudo bounded F−submodule (symbolically S-PS.B. F -submodule) if there exists φ(x)∈S=End(M) such that φ(x)∈ℵ for some x∈M implies that $\sqrt{{\mathrm{ann}}_{F}\left(ℵ\right)}=\sqrt{{\mathrm{ann}}_{F}\left(\varphi \left(x\right)\right)}.$

Examples 3.2 1- Let $M={\mathbb{Z}}_{2}⨁{\mathbb{Z}}_{4}$ as a ${\mathbb{Z}}_{8}-\text{module}$ and $ℵ={\mathbb{Z}}_{2}⨁\left\langle \bar{2}\right\rangle .$ Define φ:M⟶M by $\varphi \left(\bar{a},\bar{b}\right)=\left(\bar{a},\bar{2}\right),\forall \left(\bar{a},\bar{b}\right)\;\in \;M.$ If $\left(\bar{0},\bar{2}\right)\;\in \;M$ then $\varphi \left(\bar{0},\bar{2}\right)=\left(\bar{0},\bar{2}\right)\;\in \;ℵ$ . Therefore, $\sqrt{{\mathrm{ann}}_{{\mathbb{Z}}_{8}}\left(ℵ\right)}=\sqrt{{\mathrm{ann}}_{{\mathbb{Z}}_{8}}\varphi \left(\bar{0},\bar{2}\right)},$ and hence ℵ is S-PS.B. ${\mathbb{Z}}_{8}-$ submodule of M.

2- Consider $M=\mathbb{Z}⨁{\mathbb{Z}}_{2}$ as a ℤ−module and $ℵ=2\mathbb{Z}⨁\left\langle \bar{0}\right\rangle$ , then there exists φ:M⟶M defined by $\varphi \left(a,\bar{b}\right)=\left(a,\bar{0}\right),\forall \left(a,\bar{b}\right)\;\in \;M$ clearly, φ∈End(M). Now, suppose that $x=\left(2,\bar{1}\right)\;\in \;M$ . Sequently, $\varphi \left(x\right)=\varphi \left(2,\bar{1}\right)=\left(2,\bar{0}\right)\;\in \;ℵ$ . Thus $\sqrt{{\mathrm{ann}}_{\mathbb{Z}}\left(ℵ\right)}=\sqrt{{\mathrm{ann}}_{\mathbb{Z}}\varphi \left(2,\bar{1}\right)}=\sqrt{\left\langle \bar{0}\right\rangle }.$ Therefore, ℵ is S-PS.B. ℤ− submodule of M.

3- Suppose that ${\mathbb{Z}}_{6}$ as a ℤ−module and $M={\mathbb{Z}}_{6},ℵ=\left\langle \bar{3}\right\rangle$ . An endomorphism φ:M⟶M defined by $\varphi \left(\bar{x}\right)=\bar{0},\forall \;\bar{x}\;\in \;{\mathbb{Z}}_{6},\varphi \left(\bar{x}\right)\;\in \;ℵ\;$ if $\bar{x}=\bar{2}\;\in \;{\mathbb{Z}}_{6}$ , we have $\mathbb{Z}=\sqrt{{\mathrm{ann}}_{\mathbb{Z}}\varphi \left(\bar{2}\right)}=\sqrt{{\mathrm{ann}}_{\mathbb{Z}}\left(\bar{0}\right)}\neq \sqrt{{\mathrm{ann}}_{\mathbb{Z}}\left\langle \bar{3}\right\rangle }=2\mathbb{Z},\;ℵ$ is not S-PS.B. ℤ− submodule.

We establish that every Endo-R.B. is S.PS.B. submodule but the converse is not necessary true in general. If ℵ is an Endo-R.B. then there exists φ∈End(M) such that φ(x)∈ℵ,for somex∈M implies that ${\mathrm{ann}}_{F}\left(ℵ\right)={\mathrm{ann}}_{F}\varphi \left(x\right)$ by Ref. 7. But $\sqrt{{\mathrm{ann}}_{F}\varphi \left(x\right)}=\left\{r\;\in \;F:r^{n}\;\in \;{\mathrm{ann}}_{F}\varphi \left(x\right),n\;\in \;{\mathbb{Z}}^{+}\right\}$ and from above equality we get $\sqrt{{\mathrm{ann}}_{F}\varphi \left(x\right)}=\sqrt{{\mathrm{ann}}_{F}\left(ℵ\right).}$ Hence, ℵ is S-PS.B. F− submodule. But the converse is not true in general for example:

Let $M={\mathbb{Z}}_{2}⨁{\mathbb{Z}}_{4}$ as ℤ−module . Define φ:M⟶M as: $\varphi \left(\bar{a},\bar{b}\right)=\left(\bar{0},\bar{b}\right),\forall \left(\bar{a},\bar{b}\right)\;\in \;M$ if we take $ℵ=\left\langle \bar{0}\right\rangle ⨁{\mathbb{Z}}_{4},\left(\bar{1},\bar{2}\right)\;\in \;M\;$ then $\varphi \left(\bar{1},\bar{2}\right)=\left(\bar{0},\bar{2}\right)\;\in \;ℵ,$ implies that $\sqrt{{\mathrm{ann}}_{\mathbb{Z}}\varphi \left(\bar{1},\bar{2}\right)}=\sqrt{{\mathrm{ann}}_{\mathbb{Z}}\left(\bar{0},\bar{2}\right)}$ = $\sqrt{2\mathbb{Z}}=2\mathbb{Z}$ and $\sqrt{{\mathrm{ann}}_{\mathbb{Z}}\left(\left\langle \bar{0}\right\rangle ⨁{\mathbb{Z}}_{4}\right)}=\sqrt{4\mathbb{Z}}=2\mathbb{Z}$ .Thus ℵ is S-PS.B. ℤ− submodule, but ${\mathrm{ann}}_{\mathbb{Z}}\left(\left\langle \bar{0}\right\rangle ⨁{\mathbb{Z}}_{4}\right)=4\mathbb{Z}$ is not equal to ${\mathrm{ann}}_{\mathbb{Z}}\varphi \left(\bar{1},\bar{2}\right)={\mathrm{ann}}_{\mathbb{Z}}\left(\bar{0},\bar{2}\right)=2\mathbb{Z}$ . Therefore ℵ is not Endo-R.B. ℤ− submodule.

Definition 3.3 A F−moduleM is said to be S-PS.B. F−module if every proper submodule of M is S-PS.B F− submodule.

Examples 3.4

1-
${\mathbb{Z}}_{p}$ as a ℤ−module is S-PS.B. ℤ−module , where p is prime, since the only proper submodule of ${\mathbb{Z}}_{p}$ is $\left\langle \bar{0}\right\rangle .$ If we define $\varphi :{\mathbb{Z}}_{2}⟶{\mathbb{Z}}_{2},\varphi \;\in \;\mathrm{End}\left({\mathbb{Z}}_{2}\right)$ as $\varphi \left(\bar{x}\right)=2\bar{x},\forall \bar{x}\;\in \;{\mathbb{Z}}_{2},$ then $\varphi \left(\bar{x}\right)\;\in \;\left\langle \bar{0}\right\rangle$ and $\sqrt{{\mathrm{ann}}_{\mathbb{Z}}\left\langle \bar{0}\right\rangle }=\sqrt{{\mathrm{ann}}_{\mathbb{Z}}\varphi \left(\bar{x}\right)}=\mathbb{Z}.$ Hence ${\mathbb{Z}}_{p}$ is S-PS.B. ℤ−module.

2- Consider ${\mathbb{Z}}_{4}$ as a ℤ−module. Define $\varphi :{\mathbb{Z}}_{4}⟶{\mathbb{Z}}_{4}$ as $\varphi \left(\bar{a}\right)=\bar{0},\forall \bar{a}\;\in \;{\mathbb{Z}}_{4}$ . If we take $M={\mathbb{Z}}_{4},ℵ=\left\langle \bar{2}\right\rangle ,$ then $\varphi \left(\bar{a}\right)\;\in \;ℵ,$ hence ${\mathbb{Z}}_{4}$ is not S-PS.B. ℤ−module, since if $\bar{a}=\bar{3}\;\in \;{\mathbb{Z}}_{4},$ then $2\mathbb{Z}=\sqrt{{\mathrm{ann}}_{\mathbb{Z}}\left(ℵ\right)}\neq \sqrt{{\mathrm{ann}}_{\mathbb{Z}}\varphi \left(\bar{3}\right)}=\mathbb{Z}.$

## 4. S-Pseudo bounded modules with some modules

In this section, many modules played a major role in getting S-Pseudo bounded module such that monoform, compressible, quasi-Dedekind module and others modules. We get more results through several relationships.

Proposition 4.1 If M is a multiplication torsion-free F−module with ( F is an integral domain), then the following statements are equivalent

(i) M is S-PS.B. F−module.

(ii) M is monoform module **.**

Proof:
(i)⟹(ii) Suppose that M is S-PS.B. F−module. Since M is a multiplication torsion-free, then M is finitely generated, by Remark (2.12) and Proposition (2.13). Therefore M is scalar by Corollary (2.10). By Proposition (2.14), every φ∈End(M) is monomorphism. Suppose that φ:M⟶M be an F−homomorphism and i:ℵ⟶M is inclusion map, then φ∘i:ℵ⟶M is monomorphism and M is monoform module.

(ii)⟹(i) Let M is monoform module implies that M is uniform prime module and every submodule ℵ≠0 of M is dense. Suppose that φ∈End(M) such that φ:M⟶M and φ(x)=tx,0≠x∈M,0≠t∈F. Since M is uniform, then tx∈ℵ. Hence ${\mathrm{ann}}_{F}\left(ℵ\right)\subseteq {\mathrm{ann}}_{F}\varphi \left(x\right)$ and thus $\sqrt{{\mathrm{ann}}_{F}\left(ℵ\right)}\subseteq \sqrt{{\mathrm{ann}}_{F}\varphi \left(x\right)}.$ Let $a\;\in \;\sqrt{{\mathrm{ann}}_{F}\varphi \left(x\right)}$ implies that $a^{n}.\varphi \left(x\right)=0,\text{for some}\;n\;\in \;{\mathbb{Z}}^{+}\;$ which implies $a^{n}.\left(\mathrm{tx}\right)=0$ and $a^{n}t\left(x\right)=0.$ Since ℵ is dense submodule and ${\mathrm{ann}}_{F}\left(M\right)$ is prime ideal of F, then $t\;\notin \;{\mathrm{ann}}_{F}\left(x\right)$ and $a^{n}\;\in \;{\mathrm{ann}}_{F}\left(x\right)={\mathrm{ann}}_{F}\left(M\right)\subseteq {\mathrm{ann}}_{F}\left(ℵ\right).$ Thus $\sqrt{{\mathrm{ann}}_{F}\left(ℵ\right)}=\sqrt{{\mathrm{ann}}_{F}\varphi \left(x\right)}$ and M is S-PS.B. F−module.

However, the condition torsion-free is suffice to prove M is monoform module.

Corollary 4.2 Let M be a torsion-free S-PS.B. F−module. Then M is monoform module.

Proof: Suppose that ℵ be any non-zero submodule of M. Since M is a torsion-free module, then ∀x∈M there exists 0≠t∈F such that tx=0, we obtain x=0. Since M is S-PS.B. F−module , then ∃φ∈End(M) such that φ:M⟶M and define as φ(x)=tx,x∈M and hence tx∈ℵ. Now, assume that 0≠x∈M, we have to show that tx≠0. Let tx=0 implies that x=0, since M is torsion-free module which is contradiction and thus tx≠0. Since ℵ is an artibrary submodule, then M is monoform module.

Proposition 4.3 If M is quasi-Dedekind S-PS.B. F−module, then M is monoform module.

Proof: It is sufficient to show that every non-zero submodule of M is dense. Assume that ℵ is any non-zero submodule of M. Define φ:M⟶M by φ(x)=tx,∀x∈M. Suppose that 0≠x∈M and tx=0 and since M is S-PS.B. F−module implies that tx∈ℵ and φ(x)=0 which means x∈kerφ. Since M is quasi-Dedekind module, then φ is monomorphism. Hence x=0 which is contradiction. Thus tx≠0 and ℵ is dense submodule.

Proposition 4.4 Every S-PS.B. F−module is retractable F−module.

Proof: Assume that ℵ is a non-zero submodule of S-PS.B. F−module M, then there exists 0≠φ∈End(M) such that φ:M⟶M and defined as φ(x)=tx,0≠x∈M,φ(x)∈ℵ and $\sqrt{{\mathrm{ann}}_{F}\left(ℵ\right)}=\sqrt{{\mathrm{ann}}_{F}\varphi \left(x\right)}.$ Let Hom(M,ℵ)=0 and i:ℵ⟶M the inclusion map. Then φ=i∘g where g:M⟶ℵ,g=0. Thus φ(x)=(i∘g)(x)=i(g(x))=0 that means $\sqrt{{\mathrm{ann}}_{F}\left(ℵ\right)}\neq \sqrt{{\mathrm{ann}}_{F}\varphi \left(x\right)}$ which is a contradiction. Hence Hom(M,ℵ)≠0∀ℵ≠0 of M. Therefore M is retractable F−module.

Conversely is not true in general, we have this example:

Consider ${\mathbb{Z}}_{4}$ as a ℤ−module and $M={\mathbb{Z}}_{4},ℵ=\left\langle \bar{2}\right\rangle .$ Define $\varphi :{\mathbb{Z}}_{4}⟶{\mathbb{Z}}_{4}$ as $\varphi \left(\bar{x}\right)=\bar{0},\forall \bar{x}\;\in \;{\mathbb{Z}}_{4},\varphi \;\in \;\mathrm{End}\left({\mathbb{Z}}_{4}\right).$ Since φ(x¯)∈ℵ,∀x¯∈ℤ4,Imφ⊆ℵ then M is retractable module. But M is not S-PS.B ℤ−module , since $2\mathbb{Z}=\sqrt{{\mathrm{ann}}_{\mathbb{Z}}\left(\bar{2}\right)}\neq \sqrt{{\mathrm{ann}}_{\mathbb{Z}}\varphi \left(\bar{1}\right)}=\mathbb{Z},$ where $\bar{1}\;\in \;{\mathbb{Z}}_{4}.$

Proposition 4.5 If M is S-PS.B. torsion-free module, then M is critically compressible module.

Proof: By Corollary (4.2) and Proposition (2.17) we get M is critically compressible.

Corollary 4.6 Let M be S-PS.B. F−module, then M is critically compressible F−module if and only if every non-zero partial endomorphism of M is monomorphism.

Proposition 4.7 If M is a duo S-PS.B. F−module, then M is fully retractable module.

Proof: Since M is S-PS.B. F−module, then there exists φ is a non-zero endomorphism of M such that φ(x)∈ℵ,x∈M and $\sqrt{{\mathrm{ann}}_{F}\left(ℵ\right)}=\sqrt{{\mathrm{ann}}_{F}\varphi \left(x\right)}$ where ℵ is a submodule of M. For every φ∈End(M) we have φ(ℵ)⊆ℵ, since M is a duo. Thus the partial endomorphism of M is not zero and 0≠φ:ℵ⟶M. Therefore M is retractable module, by Proposition (4.4) so that there exists 0≠ψ:M⟶ℵ a homomorphism. Hence ψ∘φ≠0 and thus M is fully retractable module.

Corollary 4.8 If M is a duo S-PS.B. F−module and End(M) is a domain, then M is polyform module.

Proof: Assume that M is S-PS.B. F−module then by previous proposition, M is fully retractable module. Applying Proposition (2.18) we get the result.

Proposition 4.9 Let M be an uniform S-PS.B. F−module and End(M) is a domain. Then the following statements are equivalent:

(i) M is critically compressible module.

(ii) M is polyform module.

Proof:
(i)⟹(ii) Suppose that M is critically compressible module, then M is monoform, by Corollary(4.6) and thus M is polyform.

(ii)⟹(i) Assume that M is polyform module, then Corollary (2.22), M is monoform, since M is uniform. Since M is S-PS.B. F−module , then M is retractable. By Proposition (2.16), M is critically compressible module.

Corollary 4.10 If M is S-PS.B. uniform F−module where End(M) is a domain, then the following statements are equivalent:

(i) M is fully polyform module.

(ii) M is critically compressible module.

Proof: If M is uniform, then polyform and fully polyform are equivalent, by Ref. 10.

Proposition 4.11 If M is a compressible F−module, then M is S-PS.B. F−module.

Proof: Assume that φ∈End(M) where φ:M⟶M define as φ(x)=tx,x∈M. For each submodule ℵ≠0 of M there exists h:M⟶ℵ a monomorphism map, since M is a compressible module. If φ=i∘h where i is the inclusion map, then φ(x)=(i∘h)(x)=i(h(x))=h(x)∈ℵ. Suppose that $a\;\in \;\sqrt{{\mathrm{ann}}_{F}\varphi \left(x\right)}$ implies that $a^{n}.\varphi \left(x\right)=0,\;\text{for some}\;n\;\in \;{\mathbb{Z}}^{+},\forall x\;\in \;M.$ Thus $a^{n}.h\left(x\right)=0,\forall x\;\in \;M\;$ so that $h\left(a^{n}x\right)=0$ and $h\left(a^{n}x\right)=h\left(0\right)$ which means $a^{n}x=0,\forall x\;\in \;M.$ Hence $a\;\in \;\sqrt{{\mathrm{ann}}_{F}\left(M\right)}$ and $a^{n}\;\in \;{\mathrm{ann}}_{F}\left(M\right)={\mathrm{ann}}_{F}\left(ℵ\right),$ since M is prime module. Therefore $\sqrt{{\mathrm{ann}}_{F}\varphi \left(x\right)}=\sqrt{{\mathrm{ann}}_{F}\left(ℵ\right)}$ and M is S-PS.B. F−module.

Proposition 4.12 Let M be a torsion-free multiplication F−module with ( F is an integral domain).Then M is S-PS.B. F−module if and only if M is a compressible module.

Proof: Let M be S-PS.B. F−module . By Corollary (2.10) M is a scalar module and hence every φ∈End(M) is monomorphism. Since M is retractable module by Proposition (4.4), then there exists a homomorphism 0≠f:M⟶ℵ for every submodule ℵ≠0 of M. Hence φ=i∘f is monomorphism where i is an inclusion map. Thus f is a monomorphism and hence M is a compressible module.

Conversely, applying previous proposition.

Corollary 4.13 If M is a quasi-Dedekind retractable F−module, then M is S-PS.B. F−module .

Proof: Every F−homomorphism φ∈End(M) is monomorphism, since M is a quasi-Dedekind. Hence M is a compressible module, by Proposition (2.15) and thus M is S-PS.B. F−module , by Proposition (4.11).

Corollary 4.14 If M is a finitely generated, then every uniform prime module is S-PS.B. F−module .

Proof: Since M is a finitely generated implies that M is a compressible module, by (2.23). The result is obtained by Proposition (4.11).

Proposition 4.15 If M is a quasi-Dedekind F−module and $\varphi \left(M\right)⊈\cap_{\psi \;\in \;\mathrm{Hom}\left(F,ℵ\right)}\text{kerψ}$ where φ∈Hom(M,F) and ℵ is a submodule of M, then M is S-PS.B. module.

Proof: Suppose that $\varphi \left(M\right)⊈\cap_{\psi \;\in \;\mathrm{Hom}\left(F,ℵ\right)}\text{kerψ},$ then $\exists \psi^{*}:F\to ℵ$ such that $0\neq \psi^{*}\;\circ \;\varphi \;\in \;\mathrm{Hom}\left(M,ℵ\right).$ Thus M is retractable module. Since M is a quasi-Dedekind module, then M is S-PS.B. F−module by proposition (4.13).

Proposition 4.16 Let M is a quasi-Dedekind S-PS.B. F−module. Then ${\mathrm{ann}}_{F}\left(M\right)\neq {\mathrm{ann}}_{F}\left(\frac{M}{ℵ}\right),ℵ\leq M.$

Proof: Assume that ℵ≤M and ${\mathrm{ann}}_{F}\left(M\right)={\mathrm{ann}}_{F}\left(\frac{M}{ℵ}\right).$ Hence $\left[ℵ{:}_{F}M\right]={\mathrm{ann}}_{F}\left(M\right)={\mathrm{ann}}_{F}\left(x\right),\forall x\;\in \;M,$ since M is prime. Thus for every t such that tM⊆ℵ and ty∈ℵ so that tx=0,x≠0,x,y∈M which is contradiction since M is monoform module by Proposition (2.18). Thus ${\mathrm{ann}}_{F}\left(M\right)\neq {\mathrm{ann}}_{F}\left(\frac{M}{ℵ}\right).$

Corollary 4.17 Let M is a quasi-Dedekind S-PS.B. F−module. Then M is not coprime F−module.

Proposition 4.18 Let ℵ≤M and M/ℵ be a quasi-Dedekind F−module . Then M is S-PS.B. F−module .

Proof: Suppose that φ∈End(M) and φ:M⟶M defined as φ(n)=tn,n∈M. Since M/ℵ be a quasi-Dedekind module so if we define ψ:M/ℵ→M/ℵ as ψ(x+ℵ)=tx+ℵ,∀x∈M then either ψ=0 or ψ is a monomrphism. Let ψ≠0 and n+ℵ∈Kerψ then ψ(n+ℵ)=ℵ which means tn+ℵ=ℵ. Therefore, n+ℵ=ℵ so that n∈ℵ and tn∈ℵ,∀n∈ℵ. Hence $\sqrt{{\mathrm{ann}}_{F}\left(ℵ\right)}\subseteq \sqrt{{\mathrm{ann}}_{F}\varphi \left(n\right)}$ . If ψ=0 we obtain tn+ℵ=ℵ so tn∈ℵ and also $\sqrt{{\mathrm{ann}}_{F}\left(ℵ\right)}\subseteq \sqrt{{\mathrm{ann}}_{F}\varphi \left(n\right)}.$ Now, let $a\;\in \;\sqrt{{\mathrm{ann}}_{F}\varphi \left(n\right)}$ so $a^{n}.\varphi \left(n\right)=0,\;\text{for some}\;n\;\in \;{\mathbb{Z}}^{+}$ and $a^{n}\left(\mathrm{tn}\right)=0$ then $a\;\in \;\sqrt{{\mathrm{ann}}_{F}\left(\mathrm{tn}\right)},\forall \mathrm{tn}\;\in \;ℵ.$ Thus $a\;\in \;\sqrt{{\mathrm{ann}}_{F}\left(ℵ\right)}$ and we get $\sqrt{{\mathrm{ann}}_{F}\left(ℵ\right)}=\sqrt{{\mathrm{ann}}_{F}\varphi \left(n\right)}.$

Proposition 4.19 If M is S-PS.B. F−module , then M≠tM.

Proof: Assume that M=tM and ∃φ∈End(M) such that φ:M⟶M and defined as φ(x)=tx,∀x∈M, since M is S-PS.B. F−module. Thus M is retractable by (4.4) which means Imφ⊆ℵ for every submodule ℵ≠0 of M. Therefore φ(M)⊆ℵ so that tM⊆ℵ and since M=tM implies M⊆ℵ which is contradiction. Thus M≠tM.

Proposition 4.20 Let M is S-PS.B. F−module such that 0≠φ∈End(M). Then φ is not epimorphism.

Proof: Suppose that φ∈End(M).φ≠0 Since M is S-PS.B. F−module, then M is retractable module ad there exists a homomorphism 0≠f:M⟶ℵ, for every submodule ℵ≠0 of M. Put φ=i∘f:M⟶M where i:ℵ⟶M is the inclusion map. Therefore φ is not epimorphism.

Proposition 4.21 Let M be S-PS.B. torsion-free F−module . Then M is an injective.

Proof: Since M a torsion-free and S-PS.B. F−module , so that M is a monoform module by Corollary (4.2) and thus there exists a non-zero monmorphism g:ℵ⟶M defined by g(y)=y,∀y∈ℵ. Since M is S-PS.B. F−module, then M is retractable and there exists a homomorphism 0≠f:M⟶ℵ defined by f(y)=y,∀y∈M. Moreover we consider the diagram

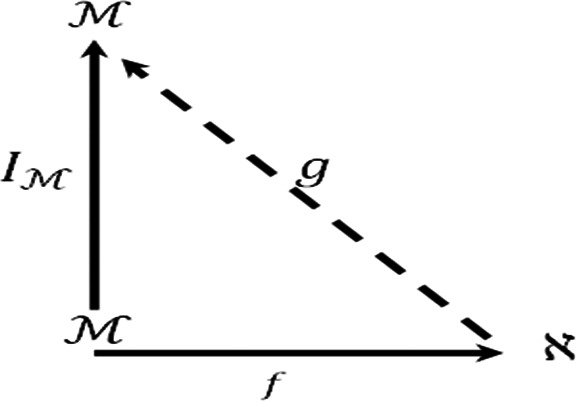

Suppose that y∈M, then $\left(g\;\circ \;f\right)\left(y\right)=g\left(f\left(y\right)\right)=g\left(y\right)=I_{M}$ . Thus $g\;\circ \;f=I_{M}$ and the diagram is commutative so M is an injective F−module.

Proposition 4.22 If M is a torsion-free and S-PS.B. F−module, then End(M) has no zero-divisors.

Proof: Suppose that φ,ψ∈End(M) are two non-zero homomorphism implies that $\exists n,n^{\prime }\;\in \;M$ such that $\varphi \left(n\right)=x\neq 0,\psi \left(n^{\prime }\right)=y\neq 0$ where x,y∈M. Since M is monoform by (4.2) and thus M is quasi-Dedekind module. Hence φ,ψ∈ are two F− monomorphisms. Thus $\left(\varphi \;\circ \;\psi \right)\left(n^{\prime }\right)=\varphi \left(y\right)\neq 0$ and (ψ∘φ)(n)=ψ(x)≠0 which means End(M) has no zero-divisors.

Proposition 4.23 If M is torsion-free S-PS.B., then M is a Rickart F−module.

Proof: Since M is an injective, by Proposition (4.21) and hence M is prime module since every torsion-free S-PS.B. F−module is monoform. By Proposition (2.20) we obtain that M is a Rickart.

Proposition 4.24 Let M be an indecomposable S-PS.B. F−module with M is N-Rickart. Then M is quasi-Dedekind.

Proof: Suppose that M is S-PS.B. F−module , then M is a retractable module, by (4.4) and thus Hom(M,ℵ)≠0 for every non-zero submodule ℵ of M. Suppose that φ:M→M is an endomorphism of M. Since M is N-Rickart, then kerφ is direct summand of M. Since M be an indecomposable so that kerφ=0. Thus φ is a F−monomorphism and M is quasi-Dedekind module.

Proposition 4.25 Let M be a torsion-free S-PS.B. Then for each φ∈End(M) there exists once φ is splits in M.

Proof: From (4.23) we get M is a Rickart F−module , Consider the following short exact sequence $0\to \text{kerφ}={\mathrm{ann}}_{M}\varphi \left(x\right)\to M\to \varphi M\to 0$ .

Consequently, kerφ is direct summand of M , since M is Rickart module. But $\text{kerφ}={\mathrm{ann}}_{M}\varphi \left(x\right)$ which means φ is splits in M.

Proposition 4.26 Let M be a S-PS.B. F−module . Then End(M) is a Rickart ring if and only if M is a Rickart module.

Proof: Since M is a S-PS.B. F−module, which means M is retractable module so that we get the result by (proposition (3.3), Ref. 16).

## 5. Conclusion

In our article, we have a new class of module called S-Pseudo Bounded and explain the relation of this module with other modules. Also, we introduced many nicely properties that join S-Pseudo Bounded with important modules such that monoform modules, retractable modules, quasi-Dedekind modules, and compressible modules. In addition, using S-Pseudo Bounded F−module as supposition lead us to get some statements that will be important for other who want to study in this field.

## Ethical considerations

This research did not involve any studies with human participants or animals and therefore did not require ethical approval.

## Data Availability

No experimental data were generated or analyzed in this study. The research is entirely theoretical within the field of pure mathematics (abstract algebra); therefore, data sharing is not applicable. No datasets were generated or analyzed during the current study. All results are theoretical and derived analytically within the framework of abstract algebra.Therefore, data sharing is not applicable to this article as no datasets were created or used.
