# Supplementary material for: New Class of S-Pseudo Bounded Modules With Some Related Concepts
Source: F1000Res. 2026 May 16;14:1385. Originally published 2025 Dec 9. [Version 3] doi: 10.12688/f1000research.172196.3 (PMC13185139; doi:10.12688/f1000research.172196.3)
Supplement: Peer review history

# Reviewer report: Reviewer response for version 3

**DOI:** 10.5256/f1000research.201107.r485381

**Published:** 2026-05-18

**Authors:** Moin A Ansari

**Affiliations:** 1 Jazan University, Jazan, Jazan, Saudi Arabia

I have gone through the revised version and found that it is well revised now. 

 I suggest to indexed this paper.

Is the work clearly and accurately presented and does it cite the current literature?

Yes

If applicable, is the statistical analysis and its interpretation appropriate?

Yes

Are all the source data underlying the results available to ensure full reproducibility?

Yes

Is the study design appropriate and is the work technically sound?

Yes

Are the conclusions drawn adequately supported by the results?

Yes

Are sufficient details of methods and analysis provided to allow replication by others?

Yes

Reviewer Expertise:

Algebras and Its Applications.

I confirm that I have read this submission and believe that I have an appropriate level of expertise to confirm that it is of an acceptable scientific standard.

# Reviewer report: Reviewer response for version 2

**DOI:** 10.5256/f1000research.198411.r476796

**Published:** 2026-05-11

**Authors:** Shanmugapriya K P

**Affiliations:** 1 Saveetha Institute of Medical and Technical Sciences (SIMATS), Thandalam, Tamil Nadu, India

The article introduces a new class of modules called SSS-pseudo bounded modules over commutative rings with identity.

 The study investigates the relationships between this new module class and several well-known modules, including monoform, quasi-Dedekind, compressible, retractable, and injective modules. Using module homomorphisms and the endomorphism ring End(M)End(M)End(M), the authors establish various properties, conditions, and corollaries related to SSS-pseudo bounded modules.

 The paper contributes to the development of module theory by extending existing algebraic concepts and providing new connections among generalized module structures. The work is mathematically relevant and may encourage further research in abstract algebra and module theory.

 Cite recent papers related to generalized module classes.

Is the work clearly and accurately presented and does it cite the current literature?

Yes

If applicable, is the statistical analysis and its interpretation appropriate?

Yes

Are all the source data underlying the results available to ensure full reproducibility?

Yes

Is the study design appropriate and is the work technically sound?

Yes

Are the conclusions drawn adequately supported by the results?

Yes

Are sufficient details of methods and analysis provided to allow replication by others?

Yes

Reviewer Expertise:

Fuzzy set, Fuzzy algebra, Interval-Valued set, Intuitionistic Fuzzy set, Neutrosophic set, Cubic set

I confirm that I have read this submission and believe that I have an appropriate level of expertise to confirm that it is of an acceptable scientific standard.

# Reviewer report: Reviewer response for version 2

**DOI:** 10.5256/f1000research.198411.r476804

**Published:** 2026-05-07

**Authors:** Anjaneyulu Naik Kalavath

**Affiliations:** 1 Acharya Nagarjuna University, Guntur, Andhra Pradesh, India

Thank you for the opportunity to peer review this manuscript.

**Manuscript Title:** New Class of S-Pseudo Bounded Modules with Some Related Concepts

 The authors define a new class of modules, termed S-Pseudo Bounded (S-PS.B.) modules. The definition appears to be original, and the authors provide clear examples and counterexamples that distinguish S-PS.B. modules from related notions such as Endo-R.B. modules. The paper is well situated within the existing literature, engaging with concepts such as bounded modules, monoform modules, compressible modules, retractable modules, and Rickart modules.

 The manuscript develops more than twenty propositions and corollaries that establish relationships between S-PS.B. modules and several well-known classes of modules, including monoform, compressible, retractable, and quasi-Dedekind modules. Overall, the arguments are rigorous, and the results are of interest to researchers in the area.

 However, the paper would benefit from a broader discussion of the structural properties of S-PS.B. modules. In particular, closure properties under quotients, products, direct summands, and extensions are not addressed. At present, only finite direct sums are considered (Remark 3.5). Expanding this discussion would strengthen the contribution.

**Strengths of this paper**

1. Introduces a new and well-defined class of modules, supported by illustrative examples and counterexamples.
2. Establishes meaningful and non-trivial connections between S-PS.B. modules and several existing module classes.

**Some Revisions Required**

1. The Introduction would benefit from 2-3 sentences clarifying the motivation for S-PS.B. modules and distinguishing them more explicitly from Endo-R.B. modules.
2. Add a title to the diagram on page 8 and reference it explicitly in the text, in line with F1000Research guidelines.
3. Clarify the relationship between S-PS.B. modules and bounded or almost bounded modules.
4. A careful proofreading is recommended to correct minor typographical and grammatical issues.

Is the work clearly and accurately presented and does it cite the current literature?

Partly

If applicable, is the statistical analysis and its interpretation appropriate?

Not applicable

Are all the source data underlying the results available to ensure full reproducibility?

No source data required

Is the study design appropriate and is the work technically sound?

Partly

Are the conclusions drawn adequately supported by the results?

Partly

Are sufficient details of methods and analysis provided to allow replication by others?

Yes

Reviewer Expertise:

Pure mathematics(Algebras), Fuzzy algebraic structures, hyper structures, soft structures, intuitionistic fuzzy sets and neutrosophic fuzzy sets with decision-making

I confirm that I have read this submission and believe that I have an appropriate level of expertise to confirm that it is of an acceptable scientific standard, however I have significant reservations, as outlined above.

# Reviewer report: Reviewer response for version 2

**DOI:** 10.5256/f1000research.198411.r476800

**Published:** 2026-05-07

**Authors:** Maurice Owino Oduor

**Affiliations:** 1 Department of Mathematics, University of Kabianga, Kericho, Kericho County, Kenya

**General Comment:** There is need to improve on the grammar. The authors should use language editor to improve on the construction of sentences, use of correct words/phrases and punctuation marks.

**Abstract**

 The second sentence should read as follows: We define a new class of F- module, namely, S-Pseudo bounded module denoted by S-PS. B. F - module and introduce different approaches to relate this class with other types of well- known modules such as ...

**1. Introduction**

 Paragraph 2. Line 3. Delete 'and connected' to read: we discovered some significant relationships with other modules.

 Definition 2.3 is not clear.

 Definition 2.5 is not clear.

 Corollary is a consequence of a theorem. So, Corollary 2.10 is a consequence of which theorem?

 Remark 2.11 is not clear.

 Restructure Proposition 2.18, by replacing the word 'with' by 'where'.

 In definition 2.19, what is varphi?

 Definition 2.21 should precede Definition 2.19 and Proposition 2.20.

 Corollary 2.22 is a consequence of which theorem?

 Corollary 2.23 is a consequence of which theorem?

**3. S- Pseudo bounded modules**

 I suggest that the number of examples under 3.2 to be reduced to two.

 Remark 3.5 should be renamed Theorem 3.5 and a precise proof be given.

**4. S- Pseudo bounded modules with some modules**

 The title sounds clumsy. This section should be merged with section 3.

 Proposition 4.1. In the proof where (ii) implies (i) to read: Given that M is a monoform module, then it is a uniform prime module...

 The last statement to read: However, the condition of torsion- free is sufficient to prove that M is monoform module.

 Proposition 4.9. Replace the word 'an' with 'a'.

 Proposition 4.21. In the proof, correct the spelling of the word 'monomorphism.' Further, a proof should not begin with the word 'Since' followed by the word 'thus'.

 Proposition 4.22. Replace the word 'implies' with the phrase 'so that'.

 Proposition 4.23. In the proof, delete the word 'an.'  Further, a proof should not begin with the word 'Since' followed by the word 'thus'.

 Proposition 4.25 is not clear. The grammar in the proof is poor.

**5. Conclusion.**

 Poor grammar. To be revisited. There is need to include a section on Recommendations for further research.

Is the work clearly and accurately presented and does it cite the current literature?

Partly

If applicable, is the statistical analysis and its interpretation appropriate?

Not applicable

Are all the source data underlying the results available to ensure full reproducibility?

No source data required

Is the study design appropriate and is the work technically sound?

No

Are the conclusions drawn adequately supported by the results?

Partly

Are sufficient details of methods and analysis provided to allow replication by others?

Partly

Reviewer Expertise:

Commutative Algebra

I confirm that I have read this submission and believe that I have an appropriate level of expertise to confirm that it is of an acceptable scientific standard, however I have significant reservations, as outlined above.

# Reviewer report: Reviewer response for version 2

**DOI:** 10.5256/f1000research.198411.r476801

**Published:** 2026-05-06

**Authors:** Moin A Ansari

**Affiliations:** 1 Jazan University, Jazan, Jazan, Saudi Arabia

Some more recent works should be mentioned in the introduction part based on modules in logical algebras such as modules over JU algebras and some other structures. Mention them in introduction part and consequently add in the reference part. 

 Shift the definition of bounded modules from introduction part to Preliminaries part. 

 Revise the English once again throughout the paper. 

 After these three modifications, I recommend accepting this article for publication in F1000Research.

Is the work clearly and accurately presented and does it cite the current literature?

Yes

If applicable, is the statistical analysis and its interpretation appropriate?

Yes

Are all the source data underlying the results available to ensure full reproducibility?

Yes

Is the study design appropriate and is the work technically sound?

Yes

Are the conclusions drawn adequately supported by the results?

Yes

Are sufficient details of methods and analysis provided to allow replication by others?

Yes

Reviewer Expertise:

Algebras and Its Applications.

I confirm that I have read this submission and believe that I have an appropriate level of expertise to confirm that it is of an acceptable scientific standard, however I have significant reservations, as outlined above.

## References

[1] : Modules over JU-Algebras. *Asia Pacific Journal of Mathematics* .2026;13: 10.28924/APJM/13-5 10.28924/APJM/13-5

# Response

**Published:** 2026-05-06

**Authors:** Amal Rashid

**Affiliations:** Mathematics, College of Education for Pure Sciences, Ibn-AL-Haitham, University of Baghdad, Heet, Anbar, Iraq

Thank you for your valuable comments and constructive feedback. We sincerely appreciate your time and effort in reviewing our manuscript. All comments have been carefully considered, and the paper has been revised accordingly, incorporating your suggestions as much as possible. We will submit the revised version based on these modifications.

# Reviewer report: Reviewer response for version 2

**DOI:** 10.5256/f1000research.198411.r476803

**Published:** 2026-04-27

**Authors:** Phani Yedlapalli, Rafi Noorbhasha

**Affiliations:** 1 Shri Vishnu Engineering College for Women, Kovvada, Andhra Pradesh, India; 2 Department of Mathematics, Siddhartha Academy of Higher Education, Vijayawada, Andhra Pradesh, India

The manuscript entitled **“New Class of S-Pseudo Bounded Modules With Some Related Concepts”** presents an original and valuable contribution to the theory of modules by introducing the notion of **S-Pseudo Bounded modules** and establishing meaningful relationships with several well-known module classes. The paper is mathematically interesting, logically developed, and supported with appropriate examples, propositions, and structural results.

 The authors have satisfactorily revised the manuscript by incorporating additional properties of S-Pseudo Bounded modules, clarifying definitions, correcting notation, and addressing previous reviewer comments. These revisions have significantly improved the presentation and strengthened the overall quality of the work.

 The results are relevant to current research in module theory and may motivate further investigations in related algebraic structures. The manuscript is suitable for indexing **after careful proofreading to correct minor typographical and grammatical errors** that remain in the text.

Is the work clearly and accurately presented and does it cite the current literature?

Yes

If applicable, is the statistical analysis and its interpretation appropriate?

Yes

Are all the source data underlying the results available to ensure full reproducibility?

Yes

Is the study design appropriate and is the work technically sound?

Yes

Are the conclusions drawn adequately supported by the results?

Yes

Are sufficient details of methods and analysis provided to allow replication by others?

Yes

Reviewer Expertise:

Algebra

We confirm that we have read this submission and believe that we have an appropriate level of expertise to confirm that it is of an acceptable scientific standard.

# Reviewer report: Reviewer response for version 1

**DOI:** 10.5256/f1000research.189909.r464812

**Published:** 2026-04-02

**Authors:** Annet Kyomuhangi

**Affiliations:** 1 Busitema University, Tororo, Uganda

Authors introduce a new class of modules called S-Pseudo Bounded modules and give enough examples. They also relate this notion to existing notions such as Rickart modules, Compressible modules,Dedekind modules, Endo R.B modules, monoform and polyform modules which have been studied extensively indicating that it is an active research area.  However, for a paper to read well, I suggest that you replace A F-module with 'An' F-module.

 In Section 3,

 2. I would have loved to see more properties of  S.PS.B. modules. For instance, closure properties; under submodules, quotients, direct sums, products, summands and extensions.

 3. Relate  S.PS.B. modules with other notions such as bounded and almost bounded modules

 4. Some terms such as Compressible, critically compressible modules and a partial endomorphism need to be defined. You defined N-Rickart modules but did not specify what a rickart module is. 

 5.  The research extends the study of Endo-R.B modules as it is shown that every Endo-R.B module is  S.PS.B. With this result, most of the results in  Section 4 are a consequence of what was studied in ***A New Class of Endo-R.B Module and Its Relationship with Modules. *** For instance, Propositions 4.1-4.20 in this paper are consequences of Propositions 4.5-5.8 in *A New Class of Endo-R.B Module and Its Relationship with Modules.*

 6. In Propositions 4.18 and 4.19, you use 't' in your statements but do not indicate what it is. Could it be an element of F?

Is the work clearly and accurately presented and does it cite the current literature?

Partly

If applicable, is the statistical analysis and its interpretation appropriate?

Not applicable

Are all the source data underlying the results available to ensure full reproducibility?

No source data required

Is the study design appropriate and is the work technically sound?

Partly

Are the conclusions drawn adequately supported by the results?

Partly

Are sufficient details of methods and analysis provided to allow replication by others?

Yes

Reviewer Expertise:

Module theory

I confirm that I have read this submission and believe that I have an appropriate level of expertise to state that I do not consider it to be of an acceptable scientific standard, for reasons outlined above.

# Response

**Published:** 2026-04-02

**Authors:** Amal Rashid

**Affiliations:** Mathematics, College of Education for Pure Sciences, Ibn-AL-Haitham, University of Baghdad, Heet, Anbar, Iraq

Thank you for your valuable comments and constructive feedback. We sincerely appreciate your time and effort in reviewing our manuscript. All comments have been carefully considered, and the paper has been revised accordingly, incorporating your suggestions as much as possible. We will submit the revised version based on these modifications.

 1- We replaced A F-module with 'An' F-module.

 2- We added more  properties of  S.PS.B. modules at the end of Section 3.

 3- We can relate S.PS.B. modules with other notions such as bounded and almost bounded but it requires many conditions to reach what we want. For instance, for bounded we need every ideal I of F is semiprime.

 4- We added the definitions of Compressible, critically compressible module and a partial endomorphism in the end of section 2. The definition of Rickart module already exists in the section 2 as 2.19

 5- In Propositions 4.18 and 4.19, 't' be an element of F.
